# Hospitalization costs of COPD cases and its associated factors: an observational study at two large public tertiary hospitals in Henan Province, China

**DOI:** 10.1186/s12877-023-04087-7

**Published:** 2023-07-25

**Authors:** Chengcheng Yu, Qingyun Xia, Quanman Li, Juxiao Wu, Xiangyu Wang, Jian Wu

**Affiliations:** 1 Department of Health Management, College of Public Health, Zhengzhou University, Zhengzhou, 450001 Henan China; 2 School of Journalism and Communication, Wuhan University, Wuhan, 430072 Hubei China; 3 Beijing Chao-Yang Hospital, 8 Gongren Tiyuchang Nanlu, Chaoyang District, Beijing, 100020 China; 4 Henan Province Engineering Research Center of Health Economy & Health Technology Assessment, Zhengzhou University, Zhengzhou, 450001 Henan China

**Keywords:** COPD, Hospitalization costs, Factors, China

## Abstract

**Background:**

The increasing prevalence of Chronic Obstructive Pulmonary Disease (COPD) has imposed a considerable economic burden. However, there remains a paucity of relevant evidence regarding the hospitalization costs of COPD cases. Therefore, in this study, we aimed to assess the hospitalization costs among COPD cases and investigate the factors that contribute to their costs in Henan Province, China.

**Methods:**

We enrolled a total of 1697 cases who were discharged with a diagnosis of COPD from January 1, 2020 to December 31, 2020, into the study. Demographic and clinical characteristics of the cases were obtained from the hospital information system (HIS) of two large tertiary hospitals in Henan Province, China. The factors associated with hospitalization costs were examined using a multiple linear regression model.

**Results:**

Total hospitalization costs of 1697 COPD cases were $5,419,011, and the median was $1952 (IQR:2031). Out-of-pocket fees accounted for 43.95% of the total hospitalization costs, and the median was $938 (IQR:956). Multiple linear regression analysis revealed that hospitalization costs were higher among older cases, cases with more comorbidities, and cases with longer length of stay. Furthermore, hospitalization costs were higher in cases who paid through private expenses compared to those covered by Urban Employee Basic Medical Insurance. Additionally, we found that cases admitted through an outpatient clinic had higher hospitalization costs than those admitted through the emergency department.

**Conclusion:**

Hospitalization costs of COPD cases are substantial. Strategies to reduce hospitalization costs, such as shortening LOS, optimizing payment plans, and preventing or managing complications, should be implemented to alleviate the economic burden associated with COPD hospitalizations.

## Background

Chronic Obstructive Pulmonary Disease (COPD), the most common chronic respiratory disease, is a major global public health concern [1, 2]. According to the Global Initiative for Chronic Obstructive Lung Disease (GOLD), an estimated 391.9 million people worldwide had COPD in 2019, resulting in 3.28 million deaths [3, 4]. China is the country with the largest number of COPD cases, nearly 100 million COPD cases, and the prevalence rate was 13.7% over the age of 40 in 2018 [5]. Even worse, the incidence of COPD in China is projected to rise due to an aging population, ambient air pollution, and a high number of active smokers (300 million adults) [6, 7]. Further, it was found that COPD ranked fifth as a cause of death in 2016 and third as a cause of disability-adjusted life years in 2017, according to the Global Burden of Disease Survey (GBD) [8, 9]. The high prevalence and related disability and mortality pose significant challenges to the healthcare system in China.

COPD is associated with many comorbidities, including pulmonary artery disease, diabetes, cardiovascular disease, and malnutrition [10, 11]. COPD and its comorbidities have placed an enormous economic burden on individuals and their families. As COPD progresses, exacerbations of respiratory symptoms increase the risk of life-threatening events, resulting in the need for hospitalization and costly life-saving medications [12]. The cost of COPD hospitalization is considerable, accounting for a substantial portion of the total medical costs for COPD cases. Previous studies in China showed that the hospitalization costs account for 65.9-77% of the total costs [13, 14]. Similarly, a USA study found that hospitalization accounts for 60% of the total costs for COPD care [15]. Additionally, a recent study from northeast China revealed that hospitalization costs for COPD almost doubled from 2005 to 2015 [16]. Factors associated with high hospitalization costs in COPD cases include age, length of stay (LOS), disease severity, and complications [17, 18]. Some of these factors are self-factor of cases, while others are associated with hospital treatment strategies.

Understanding the hospitalization costs and associated factors of COPD cases is vital in estimating the direct costs of COPD and providing suggestions for reducing the economic burden on cases. This issue has been extensively researched globally [18–20], but few studies have been conducted in Henan Province, China. What is more, most of them failed in relating to the content of these two aspects simultaneously, and did not comprehensively consider the factors associated with hospitalization costs. Thus, additional research is needed in this area.

Due to the prevalence and preventable nature of COPD, accurate estimates of its hospitalization costs are crucial to identify cost-effective interventions for COPD management. Hence, we aimed to provide an up-to-date estimation of COPD hospitalization costs and investigate the associated factors of COPD using the 2020 data from two large public tertiary hospitals in Henan Province, China.

## Methods

### Data source and sample inclusion/exclusion criteria

The study was carried out in the First Affiliated Hospital of Zhengzhou University and Henan Provincial People’s Hospital, two of the largest tertiary general hospitals in Henan Province, China. Data for cases from January 1, 2020 to December 31, 2020 were extracted from the Hospital Information System (HIS), with diagnosis of COPD coded according to the International Statistical Classification of Diseases and Related Health Problems 10th Revision (ICD-10). To ensure the accuracy of ICD-10 coding, diagnoses were determined by checking the details in medical records. Criteria for inclusion in this study were as follow: (1) the main discharge diagnosis ICD-10 coding were J40 to J44; (2) age ≥ 18 years. The exclusion criterion was the presence of primary data deficits. For cases undergoing two or more hospitalizations, each hospitalization was studied as a separate case, as our research focused on COPD cases.

### Data collection

Medical record information of COPD hospitalized cases was exported from the HIS to Microsoft Excel, which included the following data: (1) demographic characteristics (gender, age, comorbidities, the type of medical insurance, the number of hospital admissions, LOS, and mode of admission). (2) hospitalization costs, encompassing total fees, out-of-pocket fees, service fees (general medical services fees, general treatment fees, nursing fees, and other service fees), diagnostic fees (laboratory fees, pathology diagnostic fees, clinical diagnostic fees, and imaging fees), treatment fees (non-surgical treatment fees, and surgical treatment fees), medical fees (traditional Chinese medication fees and western medication fees) and other fees (e.g., rehabilitation fees, blood and blood product fees, materials fees).

### Statistical methods

For categorical variables, we described them using the percentages (%). As hospitalization costs were negatively skewed distribution, we used the median and interquartile range (IQR) to describe the hospitalization costs of COPD cases. Mann-Whitney *U* test (with 2 subgroups) or Kruskal-Wallis test (with multiple subgroups) was carried out to test differences. Prior to conducting a multiple linear regression model, the hospitalization costs were log transformed and all continuous independent variables were z-standardized. The independent variables were included in the multiple linear regression, if they were statistical significant in the univariate analysis (*p* < 0.2) [21]. No collinearity between independent variables was detected, if the variance inflation factor (VIF) was lower than 10 [22]. A probability value of *p* < 0.05 was considered statistically significant in multiple linear regression.

### Exchange rate

All fees were converted to US dollars using the average exchange rate of 6.7986 RMB to USD published by the China Foreign Exchange Trade Center for the year 2020.

## Results

### Demographic and clinical characteristics of COPD cases

As shown in Table 1, a total of 1697 COPD cases were included for analysis in the current study. 1364 (80.38%) were males, 1079 (63.58%) aged 60–79 years, 850 (50.09%) paid through New Rural Cooperative Medical Scheme (NRCMS), and 1358 (80.03%) cases had more than 3 comorbidities. In terms of hospital admissions, 918 (54.10%) were admitted for the first time, and 1236 (72.83%) were admitted through the emergency department. During hospitalization, 1280 (75.43%) cases were hospitalized for more than 5 days.

**Table 1:** Demographic and clinical characteristics of COPD cases

Characteristics	*N*	(%)
**Gender**		
Male	1364	80.38
Female	333	19.62
**Age(years)**		
< 50	82	4.83
50–59	274	16.15
60–69	553	32.58
70–79	526	31.00
≥ 80	262	15.44
**Number of comorbidities**		
< 3	339	19.97
3–4	418	24.63
5–6	797	46.97
> 6	143	8.43
**Type of medical insurance**		
Private expense	120	7.07
New Rural Cooperative Medical Scheme	850	50.09
Urban Resident Basic Medical Insurance	238	14.02
Urban Employee Basic Medical Insurance	307	18.09
Others	182	10.73
**Number of hospital admissions**		
1	918	54.10
2–3	452	26.64
≥ 4	327	19.26
**Length of stay(days)**		
< 6	417	24.57
6–8	482	28.40
9–12	382	22.51
> 12	416	24.52
**Mode of Admission**		
Emergency	1236	72.83
Outpatient clinic	453	26.69
Transfer from other medical institutions	8	0.48

### Hospitalization costs of COPD cases

Table 2 displays that the total hospitalization costs of 1697 COPD cases were $5,419,011, and the median was $1952 (IQR:2031). Out-of-pocket fees accounted for 43.95% of the total hospitalization costs, and the median was $938 (IQR:956). The hospitalization costs consisted of service fees (14.32%), diagnostic fees (24.53%), treatment fees (9.47%), medical fees (37.90%), and other fees (13.78%). The medians for these fees were $266 (IQR:335), $599 (IQR:468), $91 (IQR:237), $743 (IQR:987), and $134 (IQR:214), respectively.

**Table 2:** Composition of hospitalization costs of COPD cases

	Cost ($)	Percentage (%)	Median (IQR)
Hospitalization costs	5,419,011		1952(2031)
Out-of-pocket fess	2,381,475	43.95	938(956)
Health insurance fees	3,037,536	56.05	984(1529)
Hospitalization costs			
Service fees	775,742	14.32	266(335)
Diagnostic fees	1,329,255	24.53	599(468)
Treatment fees	513,202	9.47	91(237)
Medication fees	2,054,026	37.90	743(987)
Other fees	746,786	13.78	134(214)

IQR: interquartile range

### Univariate analysis result

The Mann-Whitney U test and Kruskal-Wallis test showed that gender (*p* = 0.919), and number of hospital admissions (*p* = 0.249) were not associated with hospitalization costs. Age group (*p* < 0.001), comorbidities (*p* < 0.001), type of medical insurance (*p* = 0.185), LOS (*p* < 0.001), and mode of admission (*p* = 0.021) were significantly associated with the hospitalization costs (*p* < 0.2) (Table 3).

**Table 3:** Univariate analysis of hospitalization costs for COPD cases

Characteristics	Hospitalization Costs ($)	
	Median (IQR)	*p*-Value
**Gender**		0.917
Male	1957(2067)	
Female	1900(1875)	
**Age(years)**		**< 0.001**
< 50	1310(1219)	
50–59	1775(1932)	
60–69	1930(1775)	
70–79	2124(2287)	
≥ 80	2172(2340)	
**Number of comorbidities**		**< 0.001**
< 3	1338(947)	
3–4	1778(1442)	
5–6	2422(2456)	
> 6	2409(2566)	
**Type of medical insurance**		**0.185**
Private expense	2142(3214)	
New Rural Cooperative Medical Scheme	1917(1863)	
Urban Resident Basic Medical Insurance	2026(2064)	
Urban Employee Basic Medical Insurance	1931(1997)	
Others	2102(2091)	
**Number of hospital admissions**		0.249
1	1913(2082)	
2–3	1961(1855)	
≥ 4	2104(1987)	
**Length of stay (days)**		**< 0.001**
< 6	1135(841)	
6–8	1543(809)	
9–12	2233(1433)	
> 12	4265(3631)	
**Mode of Admission**		**0.021**
Emergency	1937(1858)	
Outpatient clinic	2039(2525)	
Transfer from other medical institutions	4071(3485)	

### Multiple linear regression analysis

Multiple linear regression analysis showed that age group, number of comorbidities, type of medical insurance, length of stay (LOS), and mode of admission are associated with hospitalization costs (Table 4). Specifically, compared with cases under the age of 50 years, those aged 50–59 (*β* = 0.090, *p* = 0.005), 60–69 (*β* = 0.095, *p* = 0.001), 70–79 (*β* = 0.102, *p* = 0.001), and over 80 years old (*β* = 0.106, *p* = 0.001) had significantly higher costs. Compared with cases with fewer than three comorbidities, those with 3–4 (*β* = 0.057, *p* = 0.002), 5–6 (*β* = 0.150, *p*<0.001), or more than 6 comorbidities (*β* = 0.163, *p*<0.001) had significantly higher costs. Cases with LOS 6–8 (*β* = 0.135, *p*<0.001), 9–12 (*β* = 0.303, *p*<0.001), or more than 12 days (*β* = 0.572, *p*<0.001) had significantly higher costs than those under six days. Cases with Urban Employee Basic Medical Insurance (UEBMI) had lower costs (*β*=-0.068, *p* = 0.012) than those with private medical expenses. Cases admitted through the outpatient clinics had higher costs than those admitted through emergency department (*β* = 0.039, *p* = 0.009).

**Table 4:** Factors associated with the hospitalization costs in multiple linear regression analysis

Factors	Reference group	*β*	SE	*95%CI*		*p*-value
				*Lower*	*Upper*	
Age(years)						
50–59	< 50	0.090	0.032	0.028	0.151	**0.005**
60–69		0.095	0.030	0.037	0.154	**0.001**
70–79		0.102	0.030	0.043	0.161	**0.001**
≥ 80		0.106	0.032	0.043	0.170	**0.001**
**Number of comorbidities**						
3–4	< 3	0.057	0.018	0.021	0.093	**0.002**
5–6		0.150	0.017	0.117	0.183	**< 0.001**
> 6		0.163	0.027	0.110	0.216	**< 0.001**
**Type of medical insurance**						
New Rural Cooperative Medical Scheme	Private expense	-0.024	0.024	-0.072	0.024	0.334
Urban Resident Basic Medical Insurance		-0.020	0.028	-0.075	0.035	0.476
Urban Employee Basic Medical Insurance		-0.068	0.027	-0.121	-0.015	**0.012**
Others		0.007	0.029	-0.050	0.065	0.803
**Length of stay (days)**						
6–8	< 6	0.135	0.017	0.102	0.168	**< 0.001**
9–12		0.303	0.018	0.268	0.338	**< 0.001**
> 12		0.572	0.018	0.538	0.607	**< 0.001**
**Mode of Admission**						
Outpatient clinic	Emergency	0.039	0.015	0.010	0.068	**0.009**
Transfer from other medical institutions		0.045	0.089	-0.130	0.220	0.615

CI: confidence interval

## Discussion

In this cross-sectional study, we assessed COPD hospitalization costs and their components in Henan province, China, and examined the factors associated with COPD hospitalization costs. Our findings revealed that hospitalization costs increased with older age, more comorbidities, private expenses, longer LOS, and admission through outpatient clinic. As expected, health insurance significantly reduced out-of-pocket costs, with an average reimbursement rate of 56.05% for COPD cases. Our research can provide a reference for comparison with other countries and other regions in China.

The median hospitalization cost for COPD case in this study was $1,952, which is consistent with the findings of a previous study conducted in Guangdong, China [17]. One possible explanation for the similar hospitalization costs of COPD cases in Henan and Guangdong provinces could be the standardization of medical practices and treatment protocols across China, guided by centralized national guidelines for COPD management. This could have resulted in similar treatment protocols and medication use, contributing to comparable hospitalization costs. Additionally, the lack of notable variations in the severity distribution of COPD across China further supports this explanation. A recent report showed that the age-standardized disability-adjusted life year rates of COPD were relatively similar in Henan (831.31 per 100,000) and Guangdong (834.39 per 100,000) [23]. This suggests relatively consistent healthcare needs and utilization patterns in COPD cases across the two provinces, which may have also affected hospitalization costs. Finally, it is worth considering cross-regional mobility of cases seeking medical care between the two provinces as additional factors that may have contributed to the similar hospitalization costs of COPD cases.

We further found that the hospitalization costs of COPD cases in this study were different from other countries. According to previous studies [24–26], the median hospitalization cost for COPD cases in developed countries, such as the USA ($6,610), Canada ($4,936), and the Netherlands ($8,537), was significantly higher than the median cost of $1,952 observed in this study. This seems coherent since China is still a middle-income developing country with lower standards of medical facilities compared to many developed countries. However, the median of hospitalization cost was higher than that observed in some other Asian countries, such as India ($397) and Iran ($474) [20, 27]. Differences in a country’s level of economic development, healthcare policies, drug availability and infrastructure, healthcare financing, and therapeutic regimes may all contribute to the variation in hospitalization costs of COPD across countries. These factors may also explain why the costs in China, despite being lower than those in developed countries, are higher than in some other Asian countries.

In regards to the breakdown of hospitalization costs, this study found that medication fees for COPD cases accounted for 37.90%, with diagnostic fees following at 24.53%. Service fees and treatment fees accounted for only 14.32% and 9.47%, respectively. These results are consistent with previous findings in China and Turkey [17, 28]. Given the high proportion of hospitalization costs attributed to pharmacologic treatments and diagnosis in Chinese hospitals, the Chinese government has implemented several measures in recent years, including the separation of medical treatment and pharmaceutical services, aimed at reducing these fees [29]. However, despite these efforts, this study found that the proportions of diagnostic fees and medication fees remain high, while the proportion of service and treatment fees, which reflect the labor value of medical staff, remain low. Therefore, there is an urgent need to comprehensively deepen the reforms of the medical and health system to improve the labor value of medical personnel. Specific measures that can be taken include promoting the implementation of Diagnosis Related Groups (DRG) payment method reform or clinical pathway management in hospitals to standardize medical behaviors of medical staff and reduce overmedication and overdiagnosis. Additionally, hospitals can adjust charging standards and formulate charging plans based on the labor value of medical staff, such as increasing doctors’ surgical fees and reducing surgical drug fees.

The study found that older adults accounted for the majority of COPD cases and had notably higher hospitalization costs. Previous studies have reported that a significant portion of COPD-related morbidity, severity, and mortality occurs among the elderly population [30, 31]. This, in turn, has been associated with higher hospitalization costs. This is an uncontrollable factor, with underlying changes in lung function and reduced sensitivity to bronchoconstriction and hypoxia aggravating COPD with age. In addition, the older the age, the longer the course of the disease, and the possibility of repeated admissions is relatively high [32, 33]. Therefore, according to the specific conditions of COPD cases in different ages, corresponding treatment and intervention measures should be implemented to effectively improve the quality of life of cases, thereby reducing the hospitalization costs of the disease. For older COPD patients, who may have multiple comorbidities and reduced physical function, the use of pulmonary rehabilitation and technological advances, such as telemedicine and remote monitoring, may be particularly beneficial. For younger COPD patients, smoking cessation and medication management may be the primary interventions to prevent disease progression. Moreover, healthcare providers should also focus on addressing the psychosocial and behavioral aspects of COPD management, such as anxiety and depression, through patient education, counseling, and social support. Overall, a tailored approach to COPD management that considers the specific needs and preferences of each patient is essential to achieve optimal outcomes and improve their quality of life [34].

We observed higher hospitalization costs in COPD cases with multiple comorbidities. In this study, comorbidities were present in 98.2% of COPD cases, echoing findings from other studies that reported comorbidities in most COPD cases [35–37]. However, the proportion of cases with more than three comorbidities was relatively higher in this study than in some previous studies from South Korea and Spain [38, 39], which may reflect the high level of complexity of cases admitted to the two top hospitals in Henan Province, China. Comorbidities in COPD can worsen the condition, increasing the risk of morbidity and mortality [40, 41]. Apart from that, a large number of studies have reported that comorbidities contribute to more COPD-related costs for cases [42, 43]. Accordingly, it is reasonable to expect cases with more comorbidities to incur higher hospitalization costs than those with fewer or no comorbidities.

Unlike other research referred to other diseases [19, 44], this study found the hospitalization costs of the COPD cases with UEBMI was lower than those of private expense. A total of 92.9% of COPD cases were paid by medical insurance, which is basically consistent with the 97% (1.33 billion) medical insurance coverage in China in 2015 [45]. Generally speaking, medical insurance cases pay less attention to economic issues due to the reimbursement of all or part of the hospitalization costs, so use the opportunity of hospitalization to enjoy unnecessary medical services, resulting in more hospitalization costs. However, our study came to a different conclusion. On the one hand, although the proportion of reimbursement provided by UEBMI for hospitalization costs is certain, it also imposes an “out-of-pocket” threshold and a “ceiling” for insurance coverage. The out-of-pocket threshold refers to the part of the medical insurance reimbursement that needs to be borne by the case, and the reimbursement amount that does not reach this threshold will not be paid. The ceiling refers to the maximum amount of reimbursement for medical costs that has been reached or exceeded, and the subsequent medical costs will not be reimbursed. Therefore, if hospitalization costs have not reached the out-of-pocket threshold of UEBMI or have exceeded the ceiling of UEBMI, the amount paid by UEBMI for hospitalization costs may be lower than that of patients who pay by themselves. On the other hand, the medical costs also depend on factors such as the severity of the patient’s condition, the treatment method, and the treatment duration, which also affect the level of hospitalization costs. In addition, after our in-depth analysis, we found that the average surgical treatment fees of private expense cases ($148.62) is about 6.92 times that of UEBMI cases ($21.49), which reflects that some cases with private expense may have a tendency of delaying medical treatment due to lack of medical insurance reimbursement, thereby aggravating their condition. Thus, the results of this study showed that the hospitalization costs of cases with private expense were higher than that of UEBMI cases. From these results, we can conclude that increasing the scope of medical insurance coverage to enable more self-paying cases to benefit from medical insurance could be considered as a means to alleviate the problem. Examples of such measures include adjusting the deductible and the ceiling of medical insurance or expanding the coverage of medical insurance projects.

Consistent with other studies [12, 19], our study revealed a significant increase in hospitalization costs among COPD cases with longer LOS. Specifically, the median hospitalization cost for cases with LOS exceeding 12 days was approximately four times higher than that of cases with LOS less than 6 days. The elevated hospitalization costs may be attributed to several factors. Firstly, prolonged and extended LOS may worsen COPD symptoms and complications [46], leading to augmented utilization of medical resources and additional treatment fees. Secondly, longer LOS may prompt increased use of advanced medical technologies and resources [47], such as mechanical ventilation, which can substantially increase overall hospitalization costs. Finally, the larger healthcare workforce required to manage longer LOS, including specialized medical staff and support staff, can contribute to elevated healthcare fees. Reducing LOS represents a vital strategy to improve efficiency and minimize unnecessary costs for COPD cases. However, indiscriminate cost-cutting measures may increase medical risks. It is, therefore, necessary to consider the medication and diagnostic fees during hospitalization, while ensuring the quality of medical care. Based on our findings, reducing the number of hospitalization days while controlling the economic burden is crucial. Besides, we also found higher hospitalization costs for COPD cases admitted through outpatient clinic compared to those admitted through emergency department. Although COPD cases admitted through the emergency department may require more medications and rescue therapies, our study observed that cases admitted through outpatient clinic were generally older, had longer LOS, and more comorbidities. Under the combined influence of these factors, cases admitted through outpatient clinic had higher hospitalization costs than those admitted through emergency.

There are some limitations to this study. First, only hospitalization costs were analyzed, and outpatient and indirect costs were not included. Second, the data were collected from two hospitals in Henan Province, China and may not be representative of other regions or hospital types. Third, the data was limited to the period between January 1, 2020 and December 31, 2020, a relatively short time span; thus, a continuous trend comparison could not be made. Fourth, for cases undergoing two or more hospitalizations, each hospitalization was studied as a separate case, which may have led to an underestimation. Finally, data on demographic and clinical characteristics were limited (For example, COPD duration, smoking status, severity of COPD, etc., which have an impact on medical costs [48]), future studies will incorporate additional demographic and clinical characteristics to better assess hospitalization costs.

In summary, the cost of each hospitalization for COPD cases is substantial. Older age, more comorbidities, private expense, longer LOS, and admission through outpatient clinic are strongly associated with higher hospitalization costs in COPD cases, highlighting the need for targeted prevention and control measures in this population. Our study has important implications for estimating direct costs and providing suggestions for reducing the economic burden of this disease.

## Data Availability

Data are available from the corresponding author upon reasonable request with permission of the First Affiliated Hospital of Zhengzhou University and Henan Provincial People’s Hospital.

## Abbreviations

COPD: Chronic obstructive pulmonary disease
HIS: Hospital information system
LOS: Length of stay
GBD: Global burden of disease
ICD-10: International statistical classification of diseases and related health problems 10th revision
IQR: Interquartile range
CI: Confidence interval
NRCMS: New rural cooperative medical scheme
VIF: Variance inflation factor
UEBMI: Urban employee basic medical insurance
DRG: Diagnosis related groups
